# Access Sites for TAVI: Patient Selection Criteria, Technical Aspects, and Outcomes

**DOI:** 10.3389/fcvm.2018.00088

**Published:** 2018-07-17

**Authors:** Luigi Biasco, Enrico Ferrari, Giovanni Pedrazzini, Francesco Faletra, Tiziano Moccetti, Francesco Petracca, Marco Moccetti

**Affiliations:** ^1^Division of Cardiology, Fondazione Cardiocentro Ticino, Lugano, Switzerland; ^2^Division of Cardiovascular Surgery, Fondazione Cardiocentro Ticino, Lugano, Switzerland

**Keywords:** TAVI, trans-axillary, trans-aortic, trans-apical, trans-carotid, trans-septal, trans-caval, access

## Abstract

During the last ten years, transcatheter aortic valve implantation (TAVI) has become a reliable and valid alternative treatment for elderly patients with severe symptomatic aortic valve stenosis requiring valve replacement and being at high or intermediate surgical risk. While common femoral arteries are the access site of choice in the vast majority of TAVI patients, in up to 15–20% of TAVI candidates this route might be precluded due to the presence of diffuse atherosclerotic disease, tortuosity or small vessel diameter. Therefore, in order to achieve an antegrade or retrograde implant, several alterative access routes have been described, namely trans-axillary, trans-aortic, trans-apical, trans-carotid, trans-septal, and trans-caval. The aim of this paper is to give a concise overview on vascular access sites for TAVI, with a particular focus on patient's selection criteria, imaging, technical aspects, and clinical outcome.

## Introduction

Transcatheter aortic valve implantation (TAVI) has gained prime time as the preferred treatment for elderly patients suffering from severe symptomatic aortic valve stenosis and at high or intermediate risk for standard surgery (1). During the last decade, cardiac centers have faced a continuously increasing number of TAVI procedures that overtook, in some countries, the number of standard surgical aortic valve replacements (2).

Experience acquired in this setting suggests that procedural success is achieved through an accurate pre-procedural evaluation, a perfect matching between commercially available prostheses and the peculiar anatomical characteristics of TAVI patients, technical implant skills of the TAVI team and a tailored choice of access options, the latter representing one of the most critical points.

The trans-femoral access represents the preferred route in the vast majority of TAVI patients because of its minimal invasiveness and the possibility to perform the procedure under conscious sedation without intubation. Increased expertise and technical advancements lead to a significant reduction of major access site-related vascular complications that occur, nowadays, in <10% of cases.

Due to its wide diffusion and feasibility, the trans-femoral access is the preferred route in the majority of the clinical trials and is recommended as first choice by all guidelines and consensus documents (1–5).

**Figure 1:**
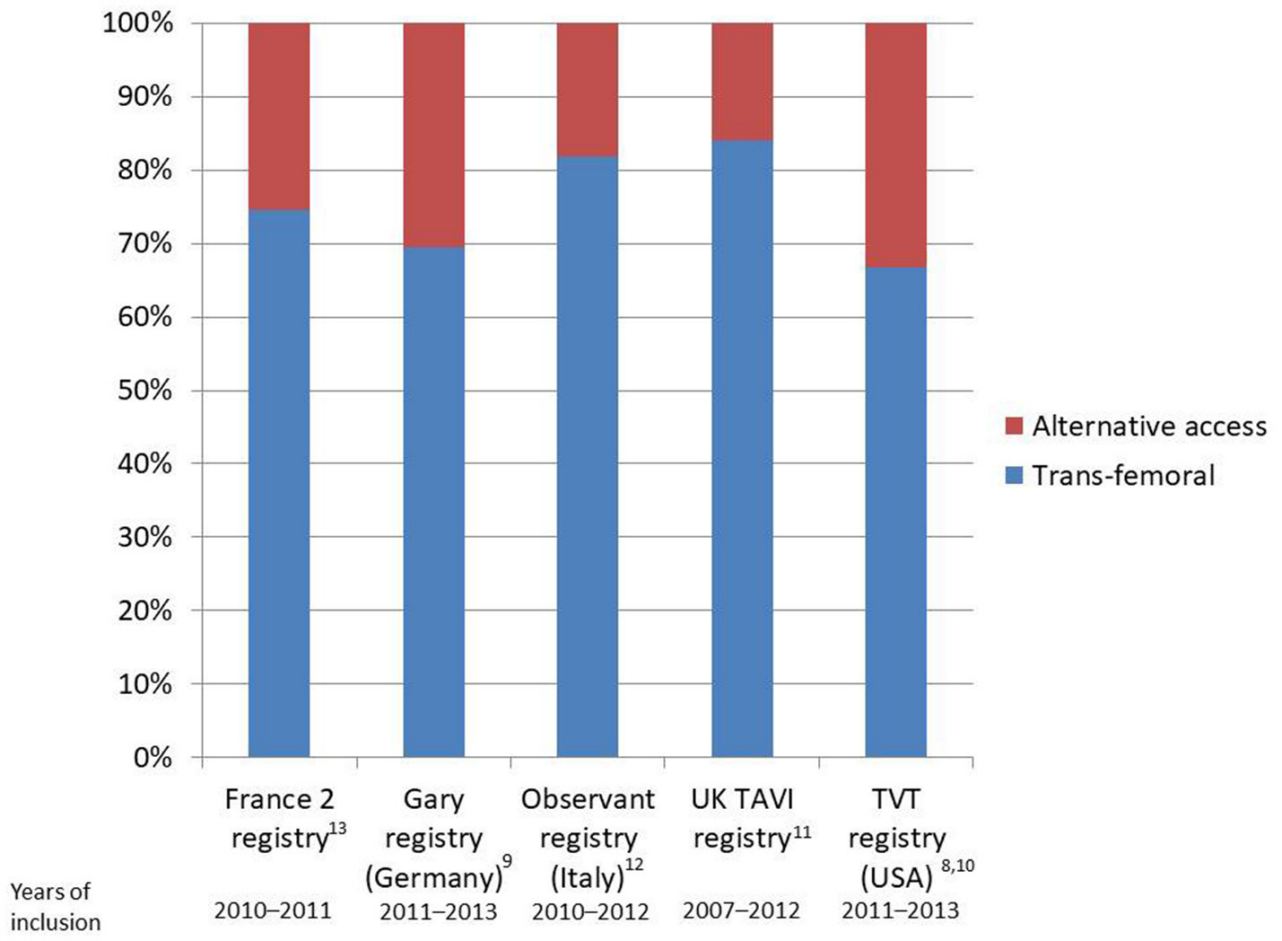
Bar plot reporting rates of transfemoral implants as compared to alternative accesses in national registries.

**Figure 2:**
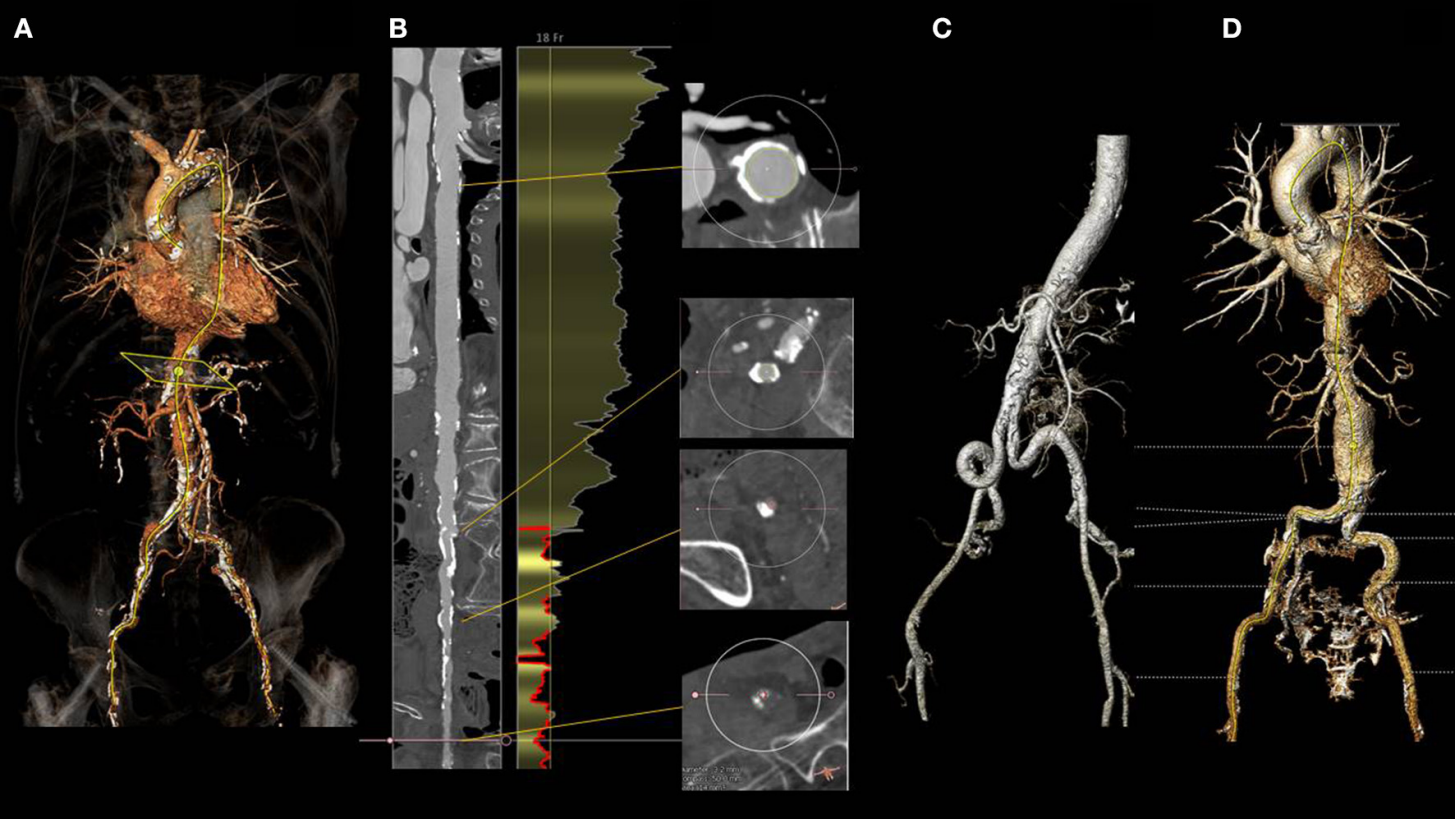
Three dimensional angio CT reconstruction obtained with the 3mensio software in a patient with severe aortic stenosis referred for TAVI **(A)**. Snake view of the aorta and right iliacofemoral arteries **(B)** clearly shows diffuse calcific atherosclerotic disease precluding trans-femoral route. **(C)** shows diffuse tortuosity of the iliac arteries, while **(D)** the incidental finding of an infrarenal abdominal aneurysm. All the above mentioned findings might preclude a transfemoral approach.

Nonetheless, data from randomized clinical trials (3–7) and registries (8–14) clearly show that the trans-femoral access might be precluded in up to 25–30% of TAVI patients due to the presence of severe arterial disease (Figure 1). In particular, obstructive peripheral vascular disease, femoro-iliac tortuosity, aortic atheromas, or the presence of previously implanted arterial grafts can seriously limit the possibility of a transfemoral access (Figure 2).

Beside technical aspects, the choice of the access in TAVI seems to be independently associated with an impact on prognosis, in particular in the case of the trans-apical approach, when manipulation of the left ventricular apex is needed (14, 15). This evidence highlights the priority of an appropriate access route selection in TAVI. So far, several alternative options for antegrade or retrograde TAVI procedures have been described, namely the trans-femoral, trans-axyllarian, trans-aortic, trans-apical, trans-carotid, trans-septal, and trans-caval. Nonetheless, no randomized comparisons are so far available, thus the choice is often based on data derived from retrospective analyses of national registries as well as on local experience.

In this report we give a concise overview on accesses for TAVI, with a particular focus on patient's selection criteria, technical aspects and clinical outcome.

## Femoral access

The common femoral artery represents the preferred access in the vast majority of TAVI procedures. This route allows a fully-percutaneous TAVI under conscious sedation/local anesthesia. Careful procedural planning and accurate choice of the proper site for vascular puncture are keys for procedural success.

### Patient's selection and planning

While obtaining the femoral access is technically easy, planning a successful procedure through this route might be demanding and time consuming. Unplanned (or angio-only guided) femoral access should be avoided whenever possible, this representing a potential risk of severe vascular complications. A detailed reconstruction of the arterial route along with precise aortic annular dimensions can be obtained at CT scan analysis, adding invaluable data about the feasibility of different approaches.

**Figure 3:**
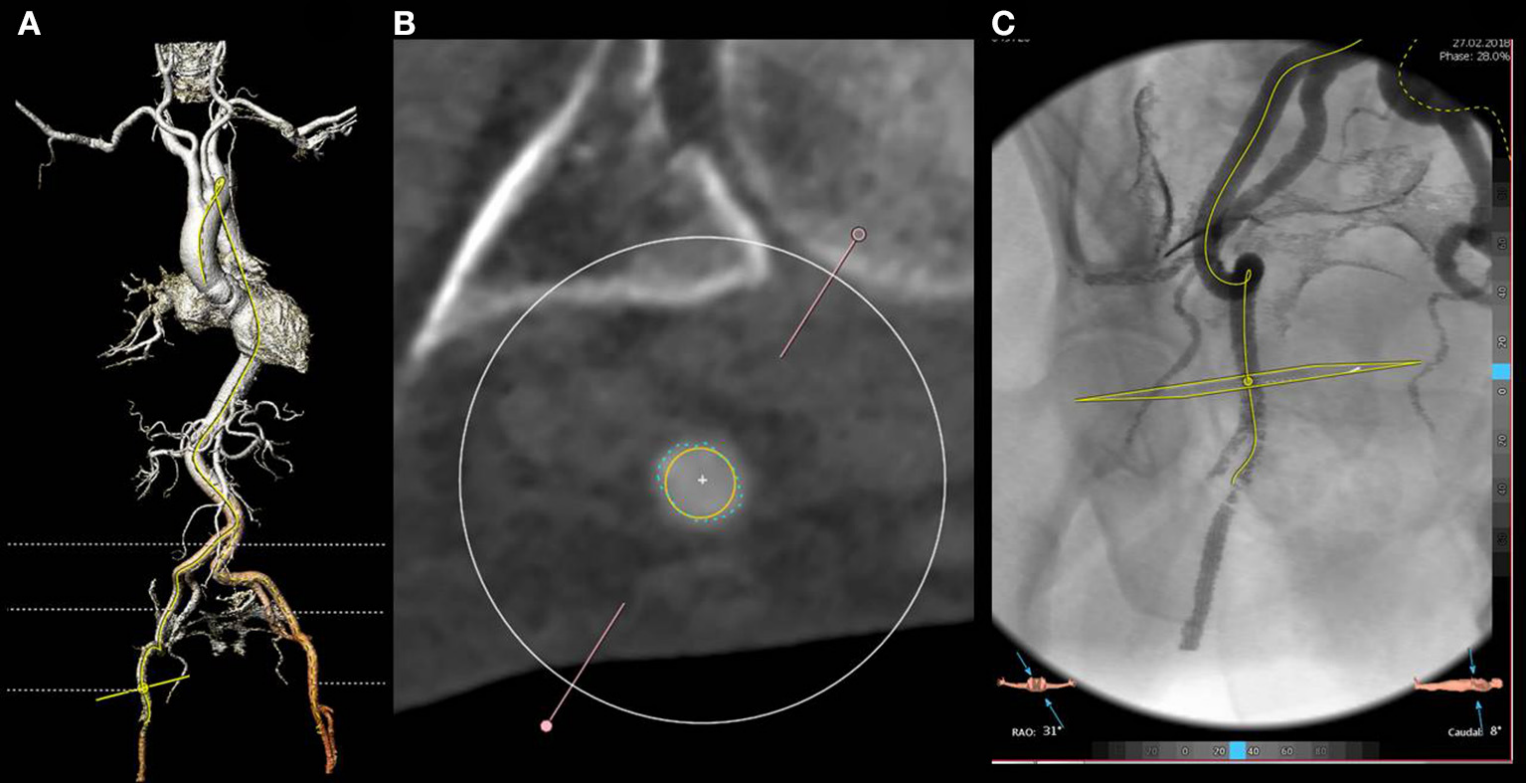
Three dimensional reconstruction of the arterial system **(A)** and cross section of the common right femoral artery at the optimal puncture site **(B)**. The artery shows a good caliber (exceeding 6.5 mm as evident from the yellow circle) and no calcifications. Moderate tortuosity of the superficial iliac artery is evident at both 3 D and angio reconstruction **(C)**. In particular, the angio reconstruction allows for the fluoro guided detection of the optimal access site, based on its relationship with the femoral head.

As a standard protocol in our center, patients referred for TAVR undergo an angio CT-scan and a 3D reconstruction extending from the aortic annulus to the superficial femoral artery with commercially available software packages (e.g., 3mensio structural Heart, Pie Medical Imaging, Maastricht, The Netherlands). The relationship of the vessels, in particular of the bifurcation, with the femoral head should be carefully evaluated. In selected cases, the angiographic reconstruction will help to perform a fluoro-guided puncture of the artery with no or minimal contrast injection (Figure 3).

While analyzing the common femoral and iliac arteries, particular attention should be paid to their caliber (that has to exceed at least 5.5 mm, ideally 6.5 mm for a 18F delivery system) and to the presence and extension of atherosclerotic plaques, calcifications as well as to the degree and extension of tortuosity. When calcifications are concentric, located anywhere from the aorto iliac bifurcation to the femoral bifurcation, even in the presence of vessels of good caliber, this could represent a potential contraindication for the trans-femoral access and the need for alternative accesses should be discussed within the Heart Team. A good estimation of the caliber of the iliac artery is of paramount importance in balloon size selection when transient occlusion is needed in bailout situations.

Beside detailed analysis of the iliiac-femoral arteries, a cautious exploration of the aorta should be performed as well in order to identify potential challenges such as tortuosity, presence of aneurysms, thrombotic appositions, or aortic arch calcifications. All these anatomic features are potential sources of embolization or causes of vascular rupture/dissection when large catheters are inserted and, therefore, can be considered as relative contraindications for a transfemoral approach.

### Technical aspects

When deemed suitable for transcutaneous access, an optimal puncture site is then identified (Figures 3A,B) in the segment of the common femoral artery extending between the inferior epigastric artery and the distal portion of the common femoral artery, ideally 1 cm above the femoral bifurcation (Figure 3C). In case of vascular complications, the most common being failure of the vascular closure device; having enough distance from the femoral bifurcation will allow the placement of a covered stent or, in alternative, a safe surgical isolation and repair.

In presence of an anterior calcification of the femoral artery, attention should be paid when percutaneous suture-based vascular closure devices (e.g., Perclose, Prostar, both from Abbot medical) are meant to be used, because their efficacy might be reduced. In those cases, surgical cut down with or without surgical endarterectomy or alternative access, should be considered.

**Figure 4:**
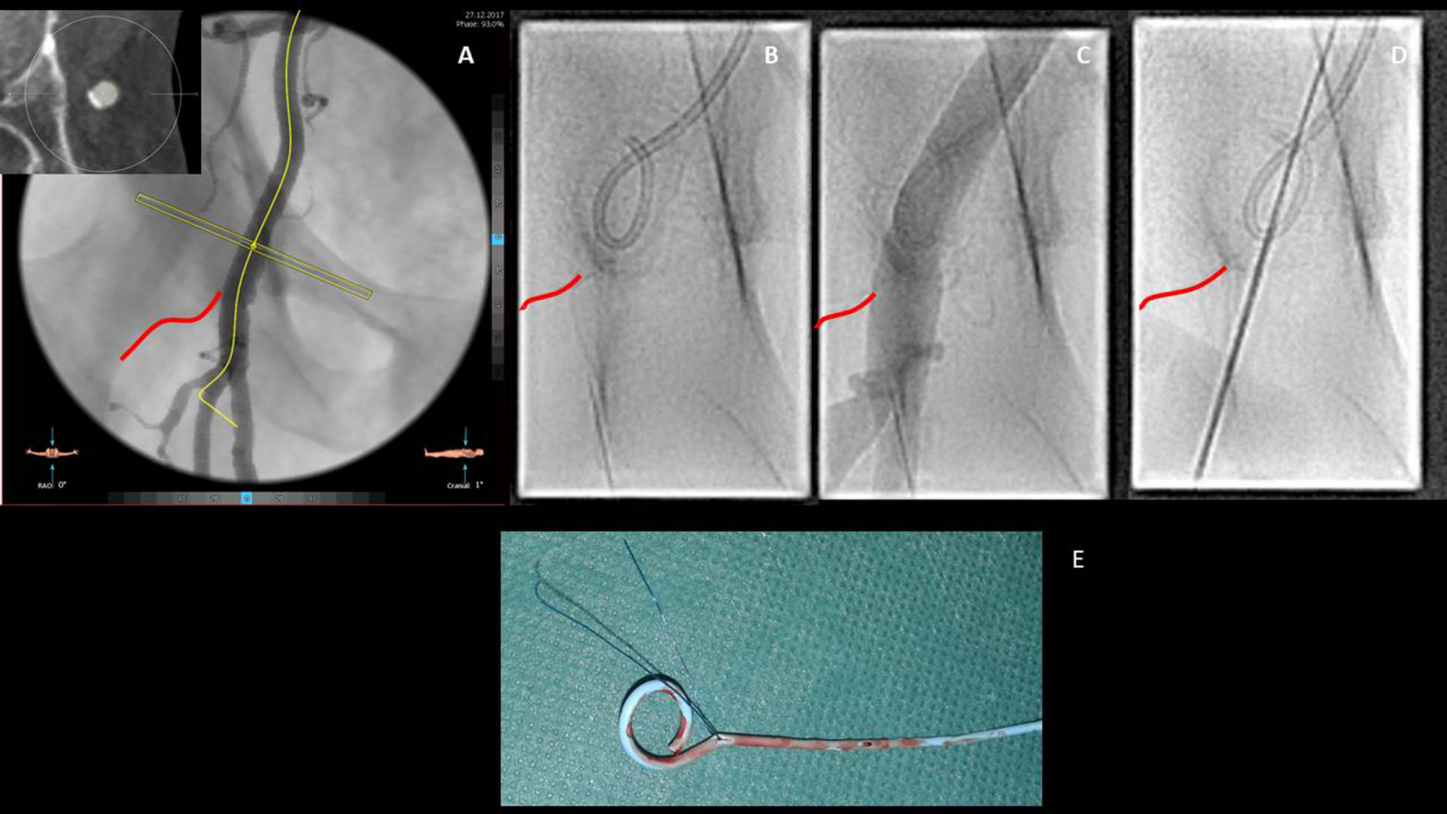
Step by step approach for the transfemoral access. Once the common femoral artery has been deemed suitable for a trans-femoral approach due to the good caliber and the lack of anterior calcifications, the relationships with the femoral head (inferior border of the femoral head is highlighted in red), observed at the angio reconstruction, have to be described **(A)**. Through a contralateral crossover, the pigtail is inserted in the common right femoral artery **(B)** and its position confirmed by contrast injection **(C)**. Vessel puncture aiming at the anterior aspect of the femoral artery is then performed **(D)**. Particular attention has to be paid in removing the pigtail before inserting the suture based closure devices with the consequent risk of catheter jailing and need for surgical removal **(E)**.

When performing a transcutaneous femoral artery puncture for TAVR, we almost invariably try to roadmap the route. For this purpose a selective angiography with a pigtail inserted by the contralateral access through a cross-over technique is used (Figures 4A–C). Then, the needle is directed toward the middle of the pigtail, and the arterial wall is punctured on its anterior aspect (Figure 4D). This will minimize the risk of vascular injury and enhance the success rate of percutaneous closure devices as well. While in the first TAVI series the Prostar was widely adopted, nowadays vascular preclosure with two Perclose/Proglide (Abbot Medical) devices inserted on the medial (2 o'clock) and lateral (11 o'clock) aspect of the arterial wall are used in the vast majority of transfemoral cases. When using this technique, attention should be paid in removing the contralateral pigtail before the insertion of the closure devices in order to prevent the entrapment of the catheter, a complication requireing surgical removal (Figure 4E). In some centers, omolateral injections using micropuncture needles (3F) are used to identify the optimal site. In obese patients with deep femoral arteries (i.e., >8 cm from the skin) as well as in cases with anterior calcifications, surgical cut down or alternative accesses should be considered. At case completion, when doubting about the efficacy of the preclosure devices implanted, a good tip to keep in mind is to perform a crossover from the contralateral femoral artery before removing the main sheath. This will allow to position a safety wire in the iliac/femoral artery that could be used rapidly for balloon occlusion and, eventually, to deliver a covered stent.

### Outcome data

Increased awareness of operators regarding the intrinsic difficulties of the femoral access in TAVI associated with technological advancements such as the progressive reduction of the caliber of the vascular sheaths and delivery systems has led to a perceivable reduction in the occurrence of major vascular complications, declined from above 10% in early PARTNER trials to about 6% in the more recent SURTAVI, NOTION and COREVALVE high risk trials (3–7). These data are in line with real-life data reported from national registries [(8–14), Table 1].

**Table 1:** Procedural outcomes according to the access site.

**Access**	**Procedural success(%)**	**30 D mortality**	**Major and life-threatening bleeding**	**Neurological events (TIA/Stroke)**	**New pacemaker implantation (%)**
Trans-femoral (3–14)	95–100	2.1–5%^‡^ 5.2–9.7%^†^	9.3–28.1%^‡^ 3.5–11.4%^†^	1.4–6.7% (30 days stroke) 2.3–4.1% (1 year stroke)	3.4–34.1 5.9–20.1
Trans-axyllarian (16)	97.9	5.7%	7.8% life threatening 36.2% major bleeding	2.1%	24.7
Trans-Aortic (17–24)	87–100	6.1–13%	0.3–12%	0–3.2%	0–14
Trans-Apical (13, 25–28)	90–96	4.6–14%	3.6–6.1%	1.3–4.1%	5.4–11.0
Trans-Carotid (29)	100	6.3%	4.2%	3.1% (all TIAs, stroke not reported)	26.5
Trans-Caval (30, 31)	100	8%	12% (6% transcaval related)	5%	16

‡ *Data derived from Partner A, Partner B, Partner II, Notion and SURTAVI trials*.

† *Data derived from TVT, Gary, UK TAVI, Observant and France2 registries*.

## Trans-axillary/trans-subclavian access

**Figure 5:**
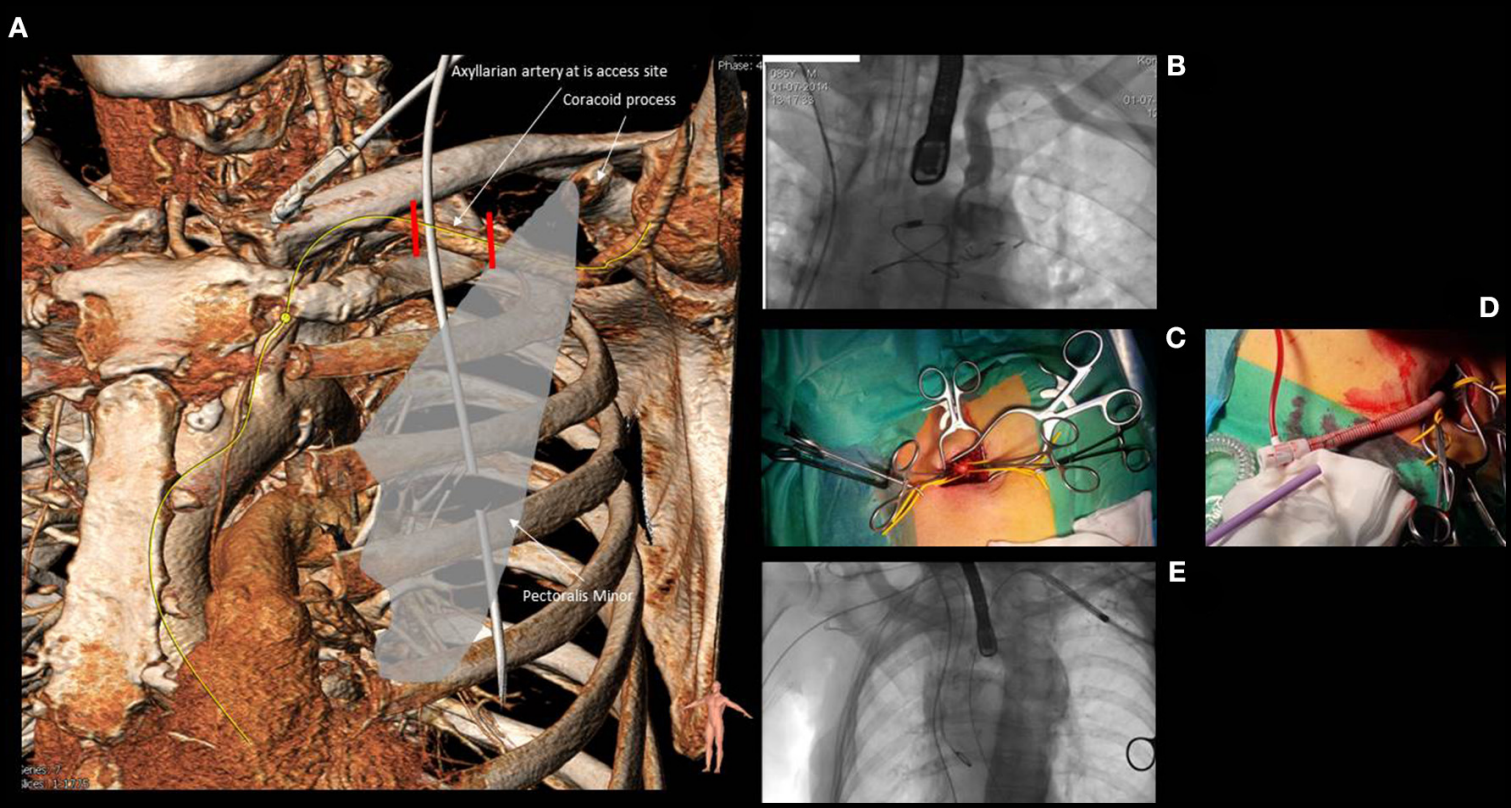
Anatomy of the subclavian and axillary artery **(A)** and its relationship with the clavicle, the first rib and the medial border of the pectoralis minor. The first segment of the axillary artery (comprised between the two red lines) is usually the target for both surgical or percutaneous approaches. **(B)** shows the angiographic anatomy of the subclavian and axillary artery. **(C)** reports the surgical cut down for axillary artery with the vessel isolated by two yellow rubber bands. **(D)** shows a “Chimney approach” performed by means of a 15 cm × 8 mm GelwaveTM prosthesis, a gelatin sealed woven polyester peripheral vascular graft and a Check-FloVR PerformerTM 18 F Cook sheath (length 35 cm), routinely used for the transfemoral modified by cutting the distal portion in order to obtain an approximate length of 10–12 cm that could allow to accommodate the sheath inside the vascular graft without extending its distal edge into the axillary artery. **(E)** shows the navigation of a Medtronic Corevalve delivery system through the left subclavian artery.

The subclavian artery is the terminal branch of the brachiocephalic artery. For the purpose of a retrograde TAVI implant, the right axillary/subclavian artery is rarely (if not ever) used due to the anatomy of the vessel leading to an unfavorable implantation angle. The left subclavian artery arises as the third branch of the aortic arch after the left common carotid artery, and exits the thorax from the superior thoracic aperture between the anterior and middle scalene muscles before passing between the first rib and the clavicle. At the lateral border of the first rib it continues as the axillary artery. The proximal third of the axillary artery (i.e., between the lateral border of the first rib and the medial border of the pectoralis minor) represents the ideal target for both surgical and percutaneous approaches (Figures 5A,B).

### Patient's selection and planning

Trans-axillary approach represents a valid option in 5–10% of patients referred for transarterial retrograde TAVI (32) and, in many centers, is considered the second option when trans-femoral TAVI is not feasible. Currently available software for CT-scan analysis allows a semi-automated 3D reconstruction of the axillary and subclavian arteries. As for the femoral approach, caliber (>6.5 mm), calcifications, tortuosity and anatomical relationships with side branches have to be taken into account. Particular attention should be paid to the aortic take-off of the subclavian artery, a typical site of atherosclerotic calcific plaque apposition.

Worth to mention is the different histological structure of the axillary and subclavian arteries when compared to the femoral artery. In fact, the subclavian, and axillary arteries are of the elastic type while the femoral is of the muscular type with a media containing smooth muscle cells instead of elastic fibers and a thicker, and more fibrous, adventitia (33). Those characteristics predispose this access to vascular complications such as ruptures or dissections. For these reasons, in the presence of a patent right internal mammarian artery to left anterior descending artery, the use of this access has been questioned due to the increased risk of vascular complication leading to the potentially lethal acute graft occlusion.

### Technical aspects

Transaxillarian approach was usually performed through a surgical cut down (Figure 5C), but the feasibility of a full percutaneous approach has been demonstrated (33).

When performing a surgical cut down, a 6–7 cm incision 1 cm below and parallel to the clavicle from the mid clavicular line to the axillary line is performed. Then, the pectoralis major muscle is dissected along its fibers, the pectoralis minor is retracted, and vessels are exposed. Attention is required to not damage to the nervous structures of the brachial plexus. Once isolated, a single or double purse string suture is placed on the subclavian artery and access to lumen is achieved by means of a direct puncture. In selected cases, a 10–12 cm Dacron vascular graft can be anastomosed end-to-side to the subclavian artery with and a standard large femoral sheath (>18F), custom modified by cutting the distal portion in order to allow to accommodate the sheath inside the vascular graft without extending its distal edge into the subclavian artery (34) (Figures 5D,E). This modified technique avoids extensive manipulation of the artery in case of borderline vascular diameter allowing a safe implantation even in patients with patent left internal mammary artery to the left anterior descending coronary artery.

A fully percutaneuos approach was described by Schäfer et al. in 2012 as the “Hamburg Sankt Georg Approach” (33). The axillary artery was landmarked with a regular J-wire and punctured below the clavicula to allow manual compression and reduce the risk of pneumothorax. Subsequently, the procedure was carried out as for the trans-femoral access. In their report, vascular complications significantly decreased when two Proglide (Abbott Vascular Devices) were used instead of a ProStar (Abbott Vascular Devices, Redwood City, California). At completion of the case, closure of the axillary artery was achieved with the pre-implanted vascular closure devices, while a peripheral balloon was always advanced in the subclavian artery to control possible bleedings.

Self-expandable prostheses are chosen in the vast majority of cases performed through this access, mainly the Medtronic Corevalve, while balloon expandable devices have been used only in selected cases.

### Outcome data

Trans-axillary approach has shown to be non-inferior to the femoral approach in terms of procedural and medium-term results in a propensity matched comparison derived from the Italian national registry (16). Both groups showed comparable rates of procedural success (subclavian 97.9 vs. femoral 96.5%, *p* = ns), major vascular complications (5.0 vs. 7.8%, *p* = ns) and life-threatening bleeding (7.8 vs. 5.7%, *p* = ns). Freedom from cardiovascular death as well as survival at 2 years was also comparable between the two groups, providing strong support to the use of this approach as a valid alternative to the trans-femoral access.

## Direct transaortic access

The direct transaortic TAVI has been originally reported by Bapat in 2012 (17, 18). The new concept behind this first report was the use of the short transapical TAVI delivery system for the retrograde TAVI implant through the ascending aorta. Since its advent, the trans-aortic technique was well accepted by heart teams and it has become a valid option in case of severe vascular disease impeding trans-femoral TAVI (19–24). Both balloon-expandable and self-expandable prostheses are currently used with good results. Some dedicated delivery systems are commercially avaliable, but standard trans-femoral systems can be used, adopting some tricks in room set-up (long delivery systems requiring good support).

### Patient's selection and planning

**Figure 6:**
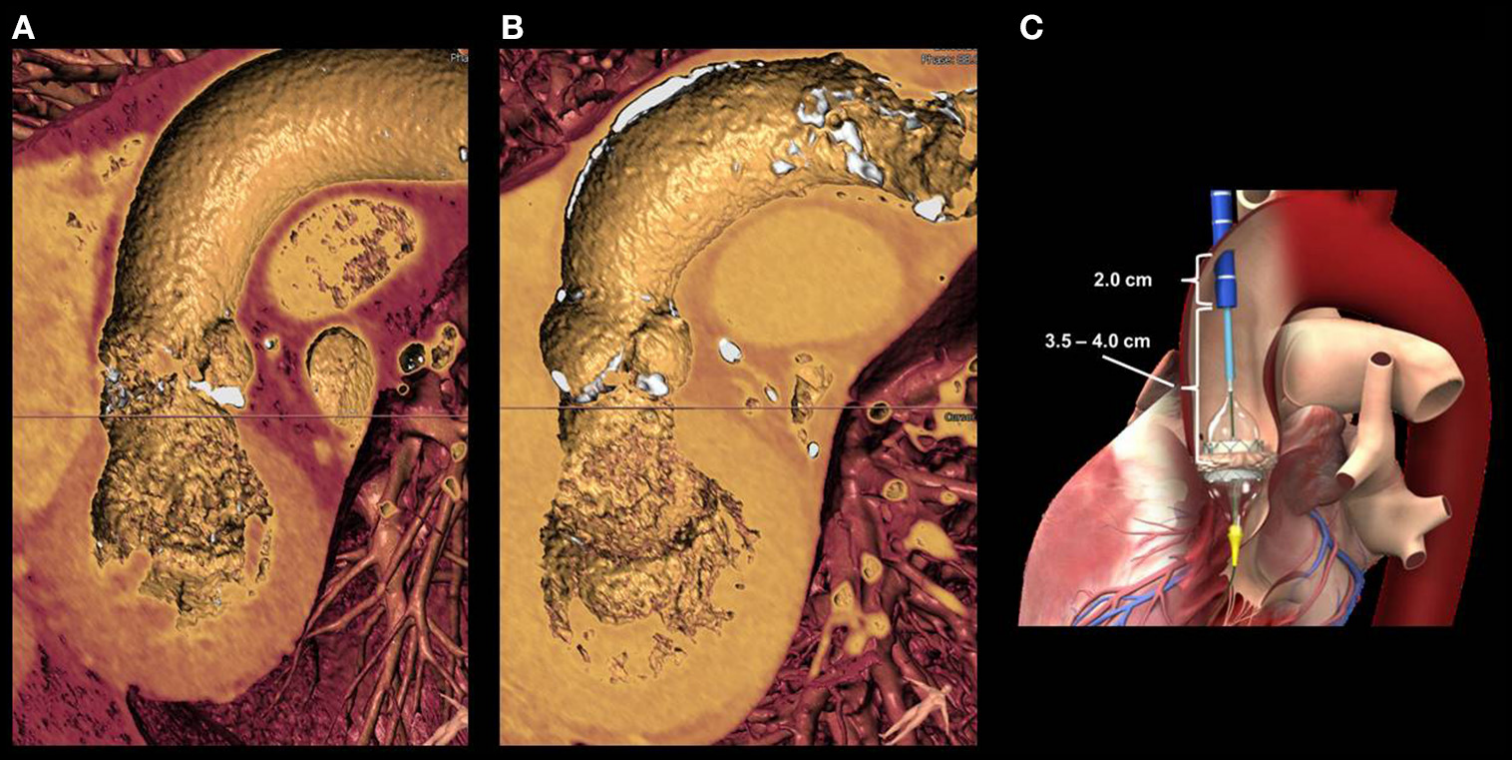
**(A)** shows a 3D reconstruction of the left ventricle, aortic valve and ascending aorta in a patient with severe aortic stenosis. Lack of anterior calcifications of the aortic walls allowed for a transaortic approach. **(B)** shows an a case of a patients with extensive anterior aortic calcifications, a potential contra-indication for trans-aortic puncture. **(C)** the distance between the aortic entry site and the aortic valve annulus is of paramount importance for the valve release. A minimal distance of 6 cm is required for small delivery systems. The larger the valve size, the longer will be the length for delivery system retrieval.

Most of the patients selected for TAVI are in fact eligible for a direct trans-aortic access with few exclusions, basically represented by the presence of thorax deformities, very short ascending aorta, porcelain aorta and the presence of a patent venous coronary artery bypass graft with proximal anastomosis on the ascending aorta at risk of damage. When facing a severely atherosclerotic aortic arch (with a good ascending portion), the direct aortic access might represent a good choice. This will allow to avoid extensive manipulation of an atherosclerotic aortic arch with the consequent risk of hembolization. In case a trans-aortic TAVI is planned, a careful evaluation of the quality of the aortic wall area where the purse string sutures will be placed (free of calcium for at least 1 square cm) is mandatory (Figures 6A,B). This is usually performed at CT-scan analysis, and for this purpose, a native contrast-free scan can be enough. Then, the trajectory between the entry site and the aortic valve annulus has to be considered in order to allow a perfect alignment between the delivery system and the native aortic valve. An “horizontal” ascending aorta (i.e., with an angle >70°) requires more banding of the delivery system with the subsequent risk of valve malalignment. Additionally, in order to allow the complete release of the valve, the aortic entry should be at least 6 cm between the above the aortic annulus (Figure 6C).

### Technical aspects

The right antero-lateral mini-thoracotomy at second intercostal space and the direct trans-aortic TAVI through an upper mini-sternotomy requires a different set-up of the cath lab as compared to the traditional trans-femoral access. First, the fluoroscopy arm is placed at patient's left side with cardiac surgeon and cardiologist standing together at the patient's right side. Compared to the mini-sternotomy, the advantage of the right mini-thoracotomy is represented by a lateral entry site into aortic lumen (right side) allowing for a straight trajectory of the delivery system through the stenotic aortic, with a consequent reduced risk of aortic damage.

Trans-aortic TAVI can be successfully performed with the Edwards Sapien balloon-expandable valve, using the dedicated trans-apical delivery system (valve mounted with the tissue skirt toward the tip of the delivery system) as well as with the Corevalve system (19–24). Other devices such as the trans-femoral Boston Lotus, the trans-femoral Accurate Neo Symetis and the St Jude Portico valve systems have been rarely used by the trans-apical route.

Due to the invasiveness of the approach, procedures are invariably performed under general anesthesia and the mechanical ventilation is obtained with a single or a double lumen endotracheal tube.

In the case the right antero-lateral mini-thoracotomy is choosen, a 5–8 cm long incision is usually performed at the right second intercostal space, parallel to the right clavicle, and muscles are gently dissected. If possible, mammary vessels should not be damaged. The pleural space is opened and the lung is either deflated (with a double-lumen tube) or displaced in order to identify the pericardium at the level of the ascending aorta. Ribs are retracted, the pericardium is opened and stay sutures are placed to expand the surgical field and pull the ascending aorta toward the operators. To identify the entry site, the ascending aorta is gently manipulated for calcium detection or a Doppler probe is used for the same purpose. The distance between the entry site and the aortic annulus is confirmed with a graduated pigtail catheter placed against the non-coronary aortic cusps under fluoroscopy. Two 3-0 or 4-0 polypropylene purse-string sutures reinforced by pledgets are placed at the entry site on the lateral wall of the ascending aorta. The ascending aorta is then punctured within the purse-string sutures and a soft guidewire is advanced toward the aortic valve, allowing for a standard valve implant. In case the trans-aortic TAVI is performed through an upper mini-sternotomy, the incision (5–8 cm) is carried out along the mid line of the thorax and the upper part of the sternum is sawed to reach the cranial portion of the ascending aorta. Once a sternal spreader is in place and the pericardium is opened, placement of the purse string sutures follows the same rules of the mini-thoracotomy but entry site is at a more more anterior. The advantage of an upper mini-sternotomy is that pleural spaces are not open. Full sternotomy is usually performed in high-risk patients requiring combined procedures such as off-pump coronary artery bypass grafting and/or tricuspid valve repair on cardiopulmonary bypass and beating heart can be considered in selected cases (35–37). The device insertion is similar to the upper mini-sternotomy.

### Clinical results

While trans-apical can be at risk of apical bleeding and major access related complications in frail elderly patients (38, 39), the transaortic TAVI can be a valid alternative in preventing apical manipulations and peripheral vascular injuries, with satisfactory clinical results. Procedural success rates of above 90% have been reported in the vast majority of series, with a 30 days mortality ranging from 6.1 to 13%. In a recently published review comparing trans-aortic vs. trans-apical TAVI procedures, Dunne et al. (40) reported similar 30-day outcomes: mortality of 7.9% (TAO) and 9.7% (TA); procedural success of 95% for both; rate of conversion to surgical aortic valve replacement of 2.1% (TAO) and 1.1% (TA); rate of new pacemaker implantation of 5.5% (TAO) and 5.9% (TA). A trend toward a lower rate of stroke in the trans-aortic TAVI group was also evident (0.9% in TAO vs 2.1% in TA).

Compared to the transapical TAVI, avoidance of apical incision with the related myocardial scar reduces the risk of apical aneurysm formation, ventricular rupture and late arrhythmias (35).

## Transapical access

The transapical access represents the historical alternative to the trans-femoral TAVI and can be performed in all patients with contraindications to the transfemoral TAVI (13, 25–28).

### Patient's selection and planning

The approach requires a left mini-thoracotomy andgeneral anesthesia. Contraindications to the transapical access route are a few, basically represented by a severely reduced left ventricular function and the presence of apical thrombus. Preoperative CT-scan images, can be useful in identifying the ventricular apex and its relationship with the thorax wall while transthoracic echocardiogram right before the procedure helps in identifying the apex and guiding the mini-thoracotomy. With regards to the commercially available transcatheter aortic valves and delivery systems, the transapical TAVI requires short dedicated delivery catheters. So far only the Edwards Sapien balloon-expandable valve and the self-expandable Symetis valve provide such possibility.

### Technical aspects

**Figure 7:**
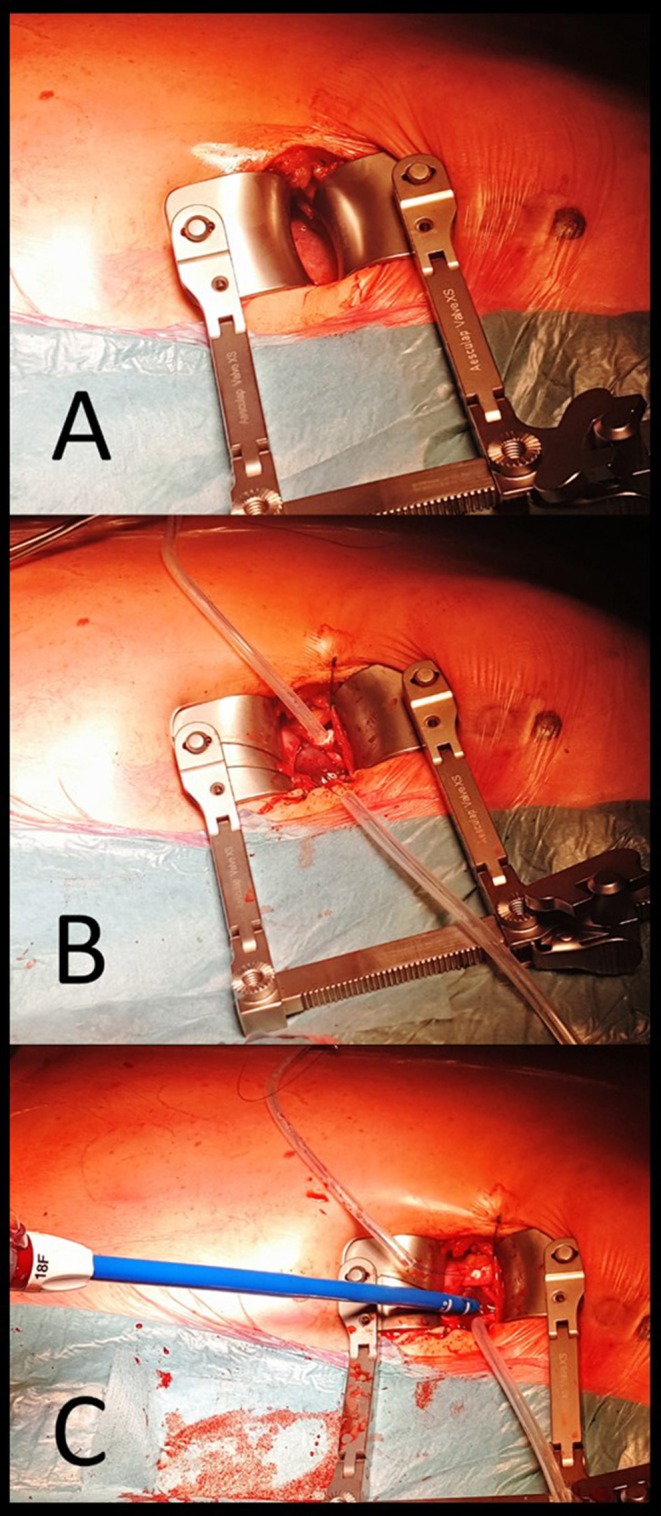
Procedural steps in trans-apical approach. **(A)** surgical incision at the left fifth intercostal space and, after placement of a rib retractor and opening pericardium the apex is exposed. **(B)** Purse-string sutures reinforced by pledgets are placed at the apex and stabilized with a Tourniquet. **(C)** after apex is punctured within the purse-string sutures and a soft guidewire is advanced with an antegrade approach toward the aortic valve and positioned in the ascending aorta. Then, a conventional 18F sheath is advanced in the left ventricular cavity.

A left antero-lateral mini-thoracotomy at the fifth intercostal space requires a different set-up of the cath lab as compared to the trans-femoral and the transaortic accesses. The fluoroscopy arm is placed at patient's right side with cardiac surgeon and cardiologist standing together at the patient's left side. Due to the invasiveness of the approach, the transapical TAVI is performed under general anesthesia and the mechanical ventilation is obtained with a single lumen endotracheal tube. A 5–8 cm long incision is performed at left fifth intercostal space and muscles are gently dissected (Figure 7A). The pleura space is opened. After the placement of a rib retractor the pericardium is opened and stay sutures are placed to expand the surgical field and pull apex toward the operators. Two concentric 3-0 or 4-0 polypropylene purse-string sutures reinforced by pledgets are placed at the apex (Figure 7B). Then, the apex is punctured within the purse-string sutures and a soft guidewire is advanced toward the aortic valve and the ascending aorta (Figure 7C). The valve is placed and delivered following standard techniques. Once implant is achieved, the delivery system and the sheath are gently removed and sutures are secured. In order to lower the intraventricular pressure during this phase, a short period of rapid pacing can be useful.

### Clinical results

The transapical TAVI can be an alternative to the transfemoral TAVI in case of severe vascular disease. Also in this case, reported procedural success rate are above 90% with 30 days mortality rate ranging from 4 to 14%.

According to data derived from the German GARY registry, trans-apical access is an independent predictor of 1 year mortality in TAVI patients. While this effect is related to the impact of apical manipulation or associated to the increased co-morbidities of those patients is still widely debated (14, 15).

## Other accesses

Starting from the original description of the first in human case of a percutaneous transcatheter implantation of an aortic valve prosthesis performed by Alain Cribier in April 2002 (41), access's choice seems to represent an intrinsic challenge of this technique. In their ground-breaking report, the procedure was performed through an antegrade approach from the right femoral vein. Access to the left atrium was obtained by trans-septal puncture, then the stenotic aortic valve was crossed on an antegrade fashion creating a venous arterial loop to allow the advancement and the stability of the percutaneous valve. Apart from this pioneering description, the antegrade transeptal approach is nowadays not considered as an option in patients unsuitable for other conventional accesses.

Rarely, the trans-carotid approach can be considered as an alternative option in patients unsuitable for trans-femoral, trans-subclavian or surgical trans-aortic/apical approaches.

While pro's are represented by avoidance of chest opening and the possibility to perform the procedure under local anesthesia, cons are mainly related to the necessity of a complex pre procedural planning with carotid and vertebral doppler to exclude significant atheromatosis, and cerebral MRI to confirm patency of the circle of Willis that could limit the cerebral perfusion during the carotid occlusion. While both common carotid arteries can be chosen, usually the left one is preferred given the straight pathway to the aortic valve. From a practical perspective, a 2 cm incision above the left clavicle allows the surgical exposure of the common carotid artery. Attention to avoid injury to the vagus nerve has to be paid. The arterial lumen is then accessed by direct puncture and surgical closure is performed at case completion. To the best of our knowledge, percutaneous access was not reported in this setting due to the complex management of potential bleedings and vascular damage. Mylotte et al. reported the feasibility and the safety of this trans-carotid approach in 96 patients enrolled in 3 different French sites (42). In their series, no major bleedings nor vascular complications related to the access site occurred, while only three transient ischemic attacks and no strokes were reported. No direct or propensity matched comparisons to trans-femoral TAVI are available so far.

The trans-caval approach, described by Greenbaum et al. in 2014 (29) is considered as the last resort in patients not qualifying for any other vascular access. Procedural planning requires a baseline CT-scan to identify a calcium free target on the right abdominal aortic wall allowing for a safe passage from the inferior vena cava to the aortic lumen of the large bore sheath. After having obtained a femoral venous access, the inferior vena cava is punctured by means of a stiff CTO wire (usually a Confianza PRO 12) mounted over a microcatheter and a standard RCA or IMA guiding catheter. The caval and aortic walls are perforated by using electrocautery applied at the distal end of the wire. Once obtained access to the aortic lumen, the wire is snared and both the microcatheter and the guiding catether are advanced into the abdominal aorta. This allows for the placement of a stiff “0.035” wire and the advancement of a large introducer sheath from the femoral vein into the aortic lumen for conventional retrograde aortic valve replacement. At case completion, heparin is reversed, and the aortic perforation is closed using a conventional vascular, duct or ventricular septal defect occluder device. The authors recently reported the 30-day outcomes of the first 100 patients from the prospective multicentre study (30). Device success, defined as successful trans-caval access and deployment of a closure device without death or emergent surgery was obtained in 98% of cases. Nonetheless, VARC-2 major or life threatening bleedings were evident in 12 patients, retroperitoneal hematomas were found at post procedural CT-scans in 24% of patients, while in 8 cases implant of an aortic covered stent was deemed necessary during the index procedure or in the early post procedural phase. Thus, so far, based on the above mentioned data, this approach should be considered as a proof of concept rather than as an effective alternative option to standard TAVI access routes and should only be considered in patients without alternative treatment options.

## Conclusions

Retrograde trans-femoral TAVI is the access of choice in the vast majority of patients with severe aortic stenosis deemed at intermediate or high operative risk for traditional surgical aortic valve replacement, and current guidelines highlight that the feasibility of a transfemoral approach should be considered as a determinant aspect favoring TAVI in the decision making process when choosing between percutaneous or surgical procedures. In line with current recommendations, also in our clinical experience, the transfemoral access is always considered as the first option. Nonetheless, in a discrete percentage of cases, this access might be precluded. This implies that several different options have been proposed as alternatives, each of them with unique features, pros and cons. The availability of different, mainly surgical accesses, should be seen as a possibility for the patients to be treated with a trans-catheter approach and for the involved heart team as a concrete opportunity to increase even more the collaboration between cardiac experts with interventional or surgical skills. So far, no randomized head to head comparisons between different access options are available, and if ever obtainable, several local factors and patient's characteristics should be considered when choosing an alternative approach. Future extension of TAVI to lower risk patients will probably result in a relative increase of transfemoral procedures. Nonetheless, in those higher risk patients in which the femoral approach is precluded, alternative routes, either percutaneously or surgically achieved might represent a concrete opportunity. When willing to avoid either general anesthesia and/or sternum/rib opening with pulmonary deflation, an option that could result as a game changer in the post op management of elderly patients allowing early mobilization and discharge, the axillary (either surgically or percutaneously performed) and trans-carotid access can be considered as a second option. On the other hand, local expertise might favor trans-aortic or trans-apical approaches due to their wide availability.

Surely, while no clear indications are still available, TAVI operators are called to a tailored decision making. Comprehensive patient's evaluation as well as extensive discussion within the heart team will represent the key points to achieve good procedural and long term outcomes.

## Author contributions

LB conceived, drafted and finalized the manuscript. EF wrote the sections regarding transapical and transaortic access. GP, FF, TM, FP, and MM critically reviewed the manuscript.

### Conflict of interest statement

EF is proctor and consultant for Edwards Lifesciences. The remaining authors declare that the research was conducted in the absence of any commercial or financial relationships that could be construed as a potential conflict of interest.
